# THE TIMING OF SUPPORT UTILIZATION AND ITS IMPLICATIONS FOR THE EFFECTIVENESS OF FORMAL CAREGIVING SUPPORT

**DOI:** 10.1093/geroni/igae098.0163

**Published:** 2024-12-31

**Authors:** Joseph Svec, Naomi Meinertz, JeongEun Lee

**Affiliations:** St. Joseph’s University, Patchogue, New York, United States; University of Missouri, Columbia, Missouri, United States; Iowa State University, Ames, Iowa, United States

## Abstract

Current literature on caregiving support indicates a mixed association between formal support (e.g., paid help, respite, training) and caregiver well-being. Compared to informal support from family and friends, formal support often comes with additional costs to caregivers in the form of financial, knowledge, and time barriers to access. This study seeks to examine those barriers, particularly the issue of when caregivers seek and utilize formal support as a predictor of its effectiveness. We utilize four waves of data from the National Study of Caregivers (NSOC) toward a series of fixed-effect linear regression models for caregiver emotional distress and physical health on formal support usage (N = 2,083). The dependent variable, experiencing any financial, emotional, or physical difficulties (M = 0.83, SD = 0.87), is regressed on whether the caregiver received support from paid help, financial help, or respite (M = 0.51, SD = 0.77). The findings show that formal support is positively associated with higher levels of caregiving difficulty (coeff = 0.22, p < 0.05); however, when controlling for past levels of difficulty (previous wave difficulty) and past usage of formal support (previous wave formal support), the positive association of formal support is mitigated. This suggests that the timing of adopting formal support can play a role in the challenges faced by caregivers, with earlier adoption potentially leading to reduced burdens of informal caregivers. While formal support remains positively associated with perceived difficulty, the mitigated coefficient when including lagged variables suggests that formal support effectiveness is also time sensitive.

